# Energy Insecurity and Cognitive Function: A Preliminary Analysis of the Offspring Study

**DOI:** 10.1093/geroni/igaf122.2292

**Published:** 2025-12-31

**Authors:** Gabriella Meltzer, Qian Li, Justina Avila-Rieger, Diana Hernández, Jennifer Manly

**Affiliations:** American University, United States; Columbia University Mailman School of Public Health, New York, New York, United States; Columbia University Irving Medical Center, New York, New York, United States; Columbia University Mailman School of Public Health, New York, New York, United States; Columbia University Irving Medical Center, New York, New York, United States

## Abstract

Older adults are highly susceptible to energy insecurity (EI), the inability to adequately meet basic household energy needs. While burgeoning research has focused on the health implications of EI for vulnerable older adults, its relationship with cognition remains unknown. This analysis examines the association between EI and cognitive health in the Offspring Study of Racial and Ethnic Disparities in Alzheimer’s Disease, which studies the risk factors of cognitive function disparities among middle-aged adults with and without parents with Alzheimer’s in the Washington Heights Inwood Columbia Aging Project parent cohort. Based on 14 indicators capturing physical, economic, and coping EI, N = 318 Offspring participants (median age 54; IQR 46, 62) were characterized as energy secure (n = 89, 28.0%) or moderately (n = 144, 45.3%), severely (n = 66, 20.8%), or extremely (n = 19, 6.0%) energy insecure. The Kruskall-Wallis rank sum test was used to compare median scores on the Selective Reminding Test (total + delayed), Animal/Vegetable Fluency test, Letter Fluency test, and Digit Span test (forward + backward) across EI categories, stratified by gender. Among men (n = 101), vegetable fluency was the lowest among those experiencing severe EI compared to those who were energy secure (10.0 vs 14.0, p = 0.045). Among women (n = 217), extreme EI was marginally associated with lower average SRT scores than the other EI groups (51 vs. 57, p = 0.059). Energy insecurity is a salient socio-environmental risk factor to consider in future studies on the burden of cognitive health disparities.

